# Attitudes and actions of asthma patients on regular maintenance therapy: the INSPIRE study

**DOI:** 10.1186/1471-2466-6-13

**Published:** 2006-06-13

**Authors:** Martyn R Partridge, Thys van der Molen, Sven-Erik Myrseth, William W Busse

**Affiliations:** 1 Department of Respiratory Medicine, Faculty of Medicine, Imperial College London, London, UK; 2 Department of General Practice, University Medical Centre Groningen (UMCG), University of Groningen, Groningen, The Netherlands; 3 European Federation of Allergy and Airways Diseases Patients' Association (EFA), Brussels, Belgium; 4 Department of Medicine, University of Wisconsin, Madison, USA

## Abstract

**Background:**

This study examined the attitudes and actions of 3415 physician-recruited adults aged ≥ 16 years with asthma in eleven countries who were prescribed regular maintenance therapy with inhaled corticosteroids or inhaled corticosteroids plus long-acting β_2_-agonists.

**Methods:**

Structured interviews were conducted to assess medication use, asthma control, and patients' ability to recognise and self-manage worsening asthma.

**Results:**

Despite being prescribed regular maintenance therapy, 74% of patients used short-acting β_2_-agonists daily and 51% were classified by the Asthma Control Questionnaire as having uncontrolled asthma. Even patients with well-controlled asthma reported an average of 6 worsenings/year. The mean period from the onset to the peak symptoms of a worsening was 5.1 days. Although most patients recognised the early signs of worsenings, the most common response was to increase short-acting β_2_-agonist use; inhaled corticosteroids were increased to a lesser extent at the peak of a worsening.

**Conclusion:**

Previous studies of this nature have also reported considerable patient morbidity, but in those studies approximately three-quarters of patients were not receiving regular maintenance therapy and not all had a physician-confirmed diagnosis of asthma. This study shows that patients with asthma receiving regular maintenance therapy still have high levels of inadequately controlled asthma. The study also shows that patients recognise deteriorating asthma control and adjust their medication during episodes of worsening. However, they often adjust treatment in an inappropriate manner, which represents a window of missed opportunity.

## Background

Asthma affects 300 million people worldwide [1], with increasing prevalence in Western Europe and the USA in particular. International guidelines stipulate goals for optimising asthma management, such as preventing chronic symptoms, minimising exacerbations and emergency care, minimising the use of rescue β_2_-agonists and maintaining normal levels of physical activity [2].

The therapeutic options available for patients with asthma depend on the severity of the condition [2-4]. Maintenance therapy with inhaled corticosteroids (ICS) is advocated for all patients with persistent asthma in order to control airway inflammation, with the addition of a long-acting β_2_-agonist (LABA) for patients who remain symptomatic on an ICS alone. ICS/LABA combination inhalers are more convenient for patients [5] and have been shown to produce effective asthma control in clinical trials [6-8].

In recent years, several patient surveys have attempted to gain insight into levels of asthma control and into patient preferences regarding asthma management [9,10]. The majority of patients in those studies, however, were selected from the general population and had mild intermittent or mild persistent asthma – and notably, not all patients had a physician-confirmed diagnosis – with over 75% of adult patients not using any regular ICS therapy. Rabe and co-workers have shown that among these patients – the majority of whom were not taking preventative therapy – asthma control is poor and does not meet guideline targets [11,12].

Studies involving patients receiving regular maintenance therapy have not been reported previously. The International Asthma Patient Insight Research (INSPIRE) study is the first multinational study to focus on patients with a physician-confirmed diagnosis of asthma who were receiving regular maintenance therapy with ICS, with or without a LABA. The study aimed to assess patients' attitudes to asthma management, levels of asthma control and the impact of the condition on patients' lives, as well as to establish the frequency and severity of worsenings and, most importantly, how patients respond to such events.

## Methods

### Study design

The study design was based on recruitment of patients by physicians, a method that provides strict controls on sampling and all other aspects of patient selection. This was a quantitative research programme utilising telephone data collection methods.

### Selection of physicians

The study was conducted between October 2004 and August 2005 in eleven countries (Australia, Belgium, Canada, France, Germany, Italy, Spain, Sweden, The Netherlands, UK and USA). Fifty physicians were recruited in each country except Germany (n = 64), Canada (n = 43) and the USA (n = 74). Primary care physicians (PCPs) and specialists (not paediatricians) were eligible, and only physicians who were either office-based or office- and hospital-based were recruited. All physicians saw a minimum number of patients each month (n ≥ 20 for PCPs; n ≥ 40 for specialists) and had not participated in asthma-related research in the 3 months before study entry. Physicians were eligible if they were aged 30–59 years and had been qualified for between 2 and 30 years. Quotas on physician type (specialist vs PCP), age, years qualified, gender and region were imposed. All physicians and patients who were recruited into the study received a payment for their time and involvement.

### Inclusion criteria

Patients were selected according to the research hypothesis, ie on the basis of their use of ICS medication either as monotherapy or in combination with another medication. Physicians were asked to identify patients with asthma who were aged ≥ 16 years, had a physician diagnosis of asthma and were attending the clinic either for a consultation or to collect a prescription for asthma medication. To minimise selection bias, physicians were requested to ask their next ten consecutive patients with asthma if they would participate in a telephone interview. This was to ensure a random selection of patients and guarantee a sufficient number of patients to undertake six successful interviews per physician. All patients had to have received a prescription for maintenance therapy with ICS, with or without LABA (including ICS/LABA combination therapy). Patients with chronic obstructive pulmonary disease were excluded but smokers were not. Physicians reported each patient's prescribed asthma medication.

All eligible patients received an invitation card. This card contained a serial number unique to each patient, which the patient was required to read out during the subsequent telephone interview. Patients were asked to sign a consent form and were informed that their details would remain strictly confidential and that information collected during the study would not be passed on to any third party, including their physician. Owing to restrictive privacy legislation in Germany, prescribing information was collected during the patient interviews.

The study was conducted in accordance with the confidentiality guidelines stipulated in the International Chamber of Commerce/European Society for Opinion and Marketing Research (ICC/ESOMAR) and European Pharmaceutical Market Research Association (EphMRA) codes of conduct, and conformed to national legislation pertaining to confidentiality and data protection in each country.

### Telephone interviews

Telephone interviews (average duration 30 min) were performed using a structured questionnaire. Interviews were conducted by local interviewers on behalf of the GfK Martin Hamblin research agency (London, UK) in the patient's native language.

The English version of the questionnaire was translated into the patient's native language, proof-read by an independent proof-reader to verify its accuracy and validated using pilot interviews.

Respondents were asked about their use of asthma therapy; need for medical intervention for asthma (defined as a visit to an emergency room, and/or hospital admission, and/or an unscheduled visit to the PCP, and/or a course of steroid tablets) in the last year; incidence of asthma worsenings (defined as occasions when asthma symptoms had become bothersome or hindering) in the past year; perceived asthma control; and activity limitations due to asthma.

### Asthma control

The Asthma Control Questionnaire (ACQ; 6-item version with forced expiratory volume in 1 s question omitted) [13,14] was used to assess asthma control. Patients were asked to recall their experiences over the past 7 days and respond to each question on a 7-point Likert scale, where 0 represents no impairment and 6 represents maximum impairment. Patients were defined as having well-controlled asthma (ACQ score < 0.75); not well-controlled asthma (ACQ score 0.75–1.5), or uncontrolled asthma (ACQ score > 1.5), as previously reported [15].

## Results

### Sample population

**Figure 1:**
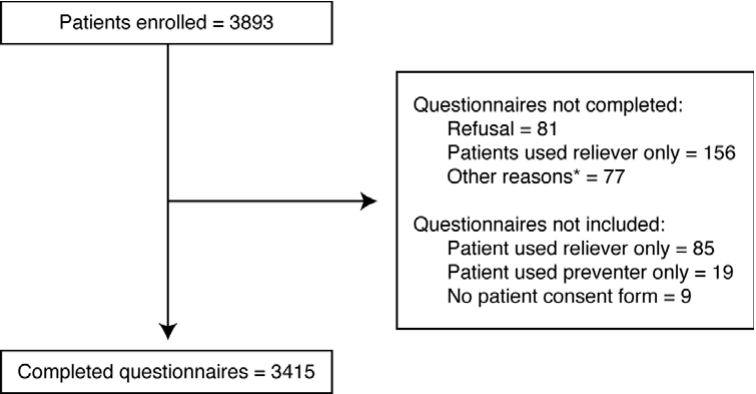
Patient flow. *Other reasons for noncompletion included: patients did not have asthma; patients were under 16 years of age; patients used preventer only; or patients had communication difficulties.

Seventy-seven per cent of the physicians recruited were PCPs and 23% were specialists. Of the 3893 patients enrolled, full interviews were completed by 3415 patients (Figure 1). Patient demographic data are shown in Table 1. The eleven national samples of asthma patients showed similar age and gender distributions, and overall results appeared to be generally very consistent across the national samples (data not shown).

Thirty per cent of patients were using ICS alone when well and the remaining 70% were prescribed ICS/LABA (61% used a combination inhaler and 9% used separate inhalers). The mean number of visits that patients had made to their physician or asthma nurse in the past year was 4.98.

### Asthma control

Despite all patients being prescribed regular ICS for maintenance therapy and a high proportion of patients using concomitant LABA, 74% of patients reported using at least one inhalation of short-acting β_2_-agonist (SABA) rescue therapy every day during the 7 days before the interview took place. Fifty-one per cent of patients reported having needed unplanned medical care (such as hospitalisation) as a result of an asthma attack on at least one occasion in the last year. Patients had had an average of 3.1 asthma-related unscheduled medical interventions in the year prior to the study.

Using overall ACQ scores to classify patients' general level of asthma control [15], 51% of patients were classified as having uncontrolled asthma, 21% had asthma that was not well controlled and only 28% of patients were classified as having well-controlled asthma [15].

**Figure 2:**
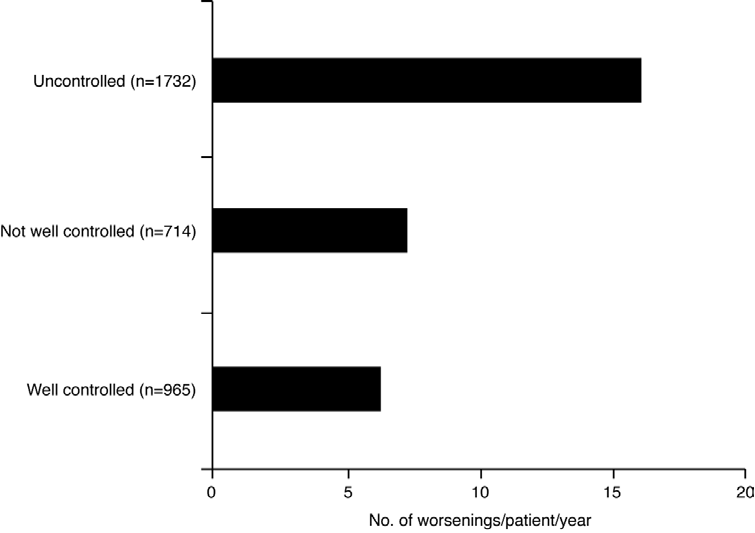
The number of worsenings experienced by patients who had had ≥ 1 worsening in the last year, by level of Asthma Control Questionnaire-defined asthma control. A worsening was defined as an occasion when asthma symptoms had become bothersome or hindering in the past year. The mean number of worsenings for the total population was 11.8/year.

Most patients (n = 3047 [89%]) experienced periods of worsenings during the last year (mean 11.8/year) (Figure 2). Even patients with well-controlled asthma reported an average of 6.3 worsenings/year. Overall, 42% of patients rated their most recent worsening as severe, 45% rated it as moderate and 13% as mild. On average, patients reported that 27% of the worsenings that they had experienced in the last year were severe.

Patients' own perceptions of how good their level of asthma control was during the week preceding the interview contrasted with the ACQ findings. Of those patients with asthma that was not well controlled according to the ACQ, 87% classed their asthma control during the week preceding the interview as "relatively good"; furthermore, 55% of patients classified as having uncontrolled asthma rated their asthma as being "relatively good".

### Asthma variability and patient response

The majority of patients (84%) recognised that they had experienced variation in their asthma during the past year. When asked whether they perceived any signs or warnings that they were about to experience a worsening of asthma, 68% of patients said that they were able to identify such signs. When described, these signs or warnings were found to correspond to asthma symptoms, the most common being "shortness of breath/getting breathless". Of the patients who reported recognising signs of an impending worsening, the mean length of time from the appearance of the first signs to the peak symptoms of the worsening was 5.1 days (range: ≤ 30 minutes to > 2 weeks). The mean length of time from peak symptoms to recovery was 6.2 days.

**Figure 3:**
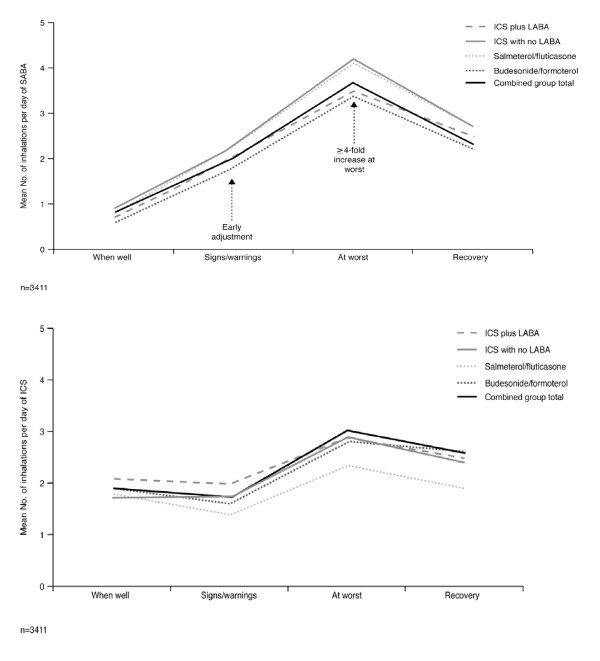
(a) Use of short-acting β_2_-agonist (SABA) rescue medication and (b) use of inhaled corticosteroid (ICS) maintenance medication during the different stages of an asthma worsening. All patients used ICS plus a separate long-acting β_2_-agonist (LABA), ICS alone, or a combination ICS/LABA product for regular maintenance therapy. Data are based on all patients who reported using each medication type at each particular stage.

Patients responded to the signs of an impending worsening by increasing their medication. Regardless of the type of maintenance therapy they were prescribed, patients reported using their SABA at the onset of symptoms, with the ICS being increased later and to a lesser extent when symptoms were at their worst (Figures 3a and 3b). A ≥ 4-fold increase in the number of SABA inhalations was reported when symptoms were at their peak compared with when patients were well. When symptoms started to decrease, patients reduced their intake of both SABA and ICS.

**Figure 4:**
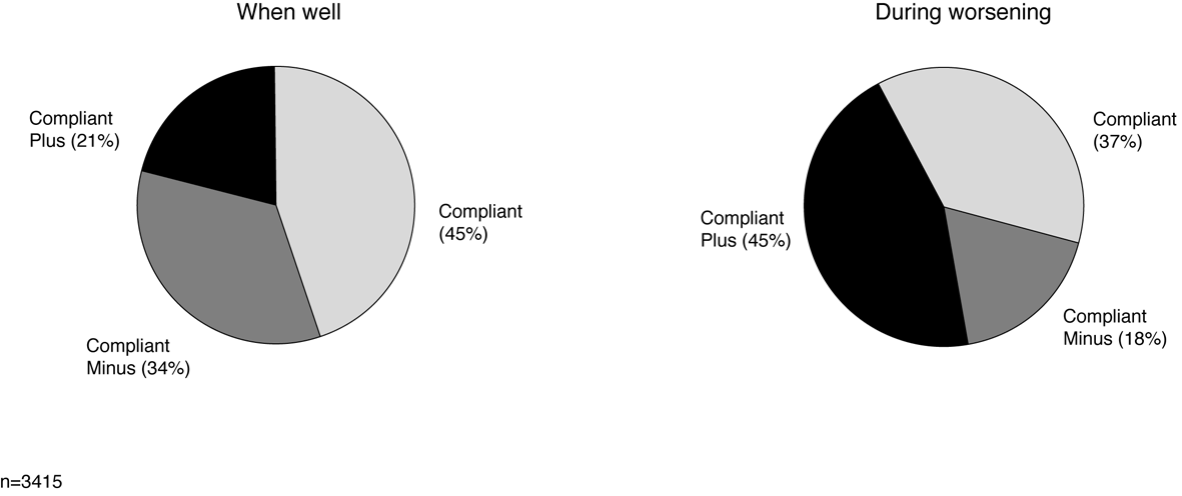
Patient compliance with their regular maintenance medication when feeling well and during asthma worsenings. Definitions were as follows: Compliant Minus: using less maintenance medication than prescribed; Compliant: using maintenance medication as prescribed; Compliant Plus: using more maintenance medication than prescribed.

Although only 29% of patients stated that their physician had instructed them to step up their maintenance medication in response to worsening asthma, 52% of patients stated that they increased the dose regardless of what they had been advised. Fewer patients underused their maintenance medication when they experienced worsening asthma compared with when they felt well (Figure 4).

### Patient attitudes to asthma management

A total of 2992 patients (88%) stated that they were very or quite confident that they could self-manage their asthma worsenings without physician visits. Many patients (n = 1858 [54%]) were concerned about taking too much medication when they felt well or during periods with no symptoms (Table 2). The majority of patients reported that they used their medication as and when necessary and were much more likely to try to manage their asthma themselves, rather than consulting their physician, when symptoms become bothersome (Table 2). Nearly 70% of patients also agreed that they preferred to adjust their maintenance ICS/combination medication to the changes in their asthma, i.e. taking less medication when well and more when their asthma worsens (Table 2). Ninety per cent of patients (n = 3075) stated that they wanted immediate relief from symptoms and 85% (n = 2898) felt confident that they knew their asthma well enough to intervene early to try to prevent a worsening of symptoms.

### Impact of asthma on patients' lives

Despite a prescription for daily maintenance therapy with ICS ± LABA, worsening asthma symptoms were found to affect all aspects of patients' daily lives, particularly their leisure and social commitments (Table 3). Over 70% of patients stated that the worst drawbacks of having asthma were the interference in their daily lives and the panic they felt as their symptoms increased (Table 4), indicating the impact of the disease on patients' health-related quality of life.

## Discussion

This study is the first to examine the perceptions of asthma among physician-recruited patients receiving daily maintenance therapy with ICS ± LABA. The results from this study emphasise the extent to which asthma management needs to improve in this group of patients.

The previously published Asthma Insights and Reality in Europe (AIRE) survey indicated that only a small proportion of patients achieve guideline targets for asthma control [11]; however, the majority of patients in that survey had mild asthma and ICS maintenance therapy was only used by 25% of adult patients [11]. In our study, we have shown that asthma control is also suboptimal in patients receiving regular maintenance therapy. Despite 70% of our patients being prescribed therapy with ICS plus LABA, only 28% had well-controlled asthma according to their ACQ scores, with 51% of patients classified as having uncontrolled asthma. Most patients (89%) experienced periods of worsenings within the last year (mean 12/year). Even patients with well-controlled asthma reported an average of 6 worsenings/year. Although one of the Global Initiative for Asthma guideline definitions of asthma control is the minimal use of as-needed β_2_-agonists [2], 74% of patients used their SABA rescue therapy every day in the week preceding the interview. Additionally, 51% of patients had required medical intervention for their asthma at least once in the past year, further demonstrating the poor level of asthma control. Theoretically, this could reflect therapy-resistant disease or an underestimation of disease severity by doctors and prescription of insufficient medication. Other possible explanations include underuse of medication by patients or their failure to recognise deteriorating asthma and adjust their medication. Regardless of the cause, a large proportion of patients had suboptimally controlled asthma.

Asthma was shown to impact on all aspects of patients' lives. In particular, patients referred to the panic or fear they have when their symptoms increase, emphasising the significant emotional burden of asthma. Asthma's interference with daily activities was reported by most patients to be the worst part of having the disease. These restrictions imposed by asthma demonstrate the extent to which poor control affects patients' health-related quality of life.

There was a discrepancy between the level of asthma control demonstrated by the ACQ questionnaire and patients' perceived level of control. These data show that even patients prescribed maintenance medication and with recent physician contact tend to underestimate their asthma symptoms and tolerate suboptimal control. This situation may reflect a lack of information from PCPs and nurses and a lack of regular contact with medical professionals [10]. Alternatively, it may be that patients consider their level of symptoms to be acceptable and are motivated to take the least "effective" dose of their medication. Indeed, patients stated that taking too much medication during periods with no symptoms was a concern. Additionally, guidelines do emphasise the need for patients to be prescribed the lowest dose of medication that controls the disease [2] and some studies suggest that overtreatment with ICS is not uncommon [16].

Most patients reported experiencing early warning signs – symptoms such as breathlessness – before periods of worsening asthma. The mean period from the occurrence of these early signs to the peak of a worsening was approximately 5 days and was followed by a recovery period of similar length. This time period from the onset of initial symptoms to an exacerbation, or worsening, is in line with observations from the FACET study. Tattersfield and co-workers [17] showed that in the FACET study, patient-reported asthma symptom scores increased and peak expiratory flow decreased in the days before an asthma exacerbation, returning to previous levels in the days after the event. Such a period of worsening symptoms is likely to represent a window of opportunity, during which patients could intervene early by increasing their asthma medication to prevent further worsening of symptoms.

Our data demonstrate that patients try to intervene early in response to signs of an impending worsening by increasing their intake of medication, and fewer patients underused their maintenance medication during such periods. However, the current study highlights a missed opportunity, as patients tended to adjust their therapy in a suboptimal manner – increasing their intake of SABA when symptoms first appear, but only increasing their ICS dose when symptoms had continued to worsen, at the peak of an asthma attack. Despite controversy regarding the benefit of increasing the dose of ICS when asthma deteriorates [18,19], an extensive review has shown that providing patients with individual written asthma action plans, with advice to double or treble the dose of ICS, can reduce symptoms and unscheduled use of healthcare resources [20]. Evidence suggests that intervening early with ICS or ICS/LABA medication provides better long-term asthma control [21-24]. Thus, the failure of patients to increase their ICS in response to early symptoms may be an important factor contributing to frequent worsenings and suboptimal asthma control. The finding that patients express an ability and willingness to alter asthma therapy themselves – but adjust the wrong medication type at the onset of worsenings – suggests failure in the implementation of the self-management education that has been recommended by guidelines for over 15 years [25].

During periods of worsening asthma, treatment regimens whereby patients increase their ICS/LABA maintenance dose in line with a self-management plan have been demonstrated to improve asthma control compared with fixed-dose combination therapy plus SABA [22,26]. A subsequent review found that stating when to increase ICS and when to start taking oral steroids are key features for inclusion in patient action plans [27]. Self-management action plans have been shown to improve asthma-specific quality of life, as patients feel less anxious about the influence of asthma on their daily activities [28] and self-management plans may also enhance patient compliance [29]. In our study, the majority of patients stated that they disliked the uncertainty associated with asthma exacerbations, but felt confident about self-managing their asthma and intervening early by increasing their medication. These results are in keeping with previous data demonstrating that patients feel comfortable adjusting their own medication without recourse to a healthcare professional [10]. From our study, it is clear that the impact of asthma on patients remains considerable, in terms of increased anxiety, dislike of uncertainty, interference with normal activities, fear of being hospitalised and fear of having attacks in front of their family (Table 4). Twenty-eight per cent of respondents described feeling stigmatised by their asthma, a proportion remarkably similar to that reported 17 years ago in a smaller interview/questionnaire-based study despite the apparent increase in public awareness of asthma over the past two decades [30].

The study recruited patients who had recently visited their physician's clinic, either for consultation or to receive a prescription. This inclusion criterion potentially excluded patients with poor access to medical care – often associated with lower socioeconomic status and increased disease morbidity [31]. As a result of excluding such patients, the level of asthma control across the globe may be worse than identified by our findings. Conversely, the inclusion of patients who had recently attended their physician's clinic may have introduced a bias towards those with more severe disease. It is also possible that some patients may have had chronic obstructive pulmonary disease rather than asthma, therefore giving the impression of more severe symptoms. However, the chances of misdiagnosis in the present study should be lower as patients were recruited by physicians rather than by telephone or direct door-to-door recruitment, as in previous studies. The intention of this study was to recruit patients typically seen in everyday practice and these results are, therefore, applicable to the practicing physician. One of the strengths of this study is the insight that it offers into patients' attitudes and actions across eleven different countries in three continents. Despite the geographical spread, the results appeared to be remarkably consistent across countries.

## Conclusion

The majority of patients with asthma receiving maintenance therapy who had had recent contact with their physician were found to have poor asthma control. Regardless of the availability of effective medication, patients' asthma continues to have a large impact on their health and health-related quality of life. This study has shown that patients recognise deteriorating asthma control and are willing to self-treat episodes of worsening. However, patients often adjust their medication in an inappropriate manner, which represents a window of missed opportunity. These findings demonstrate the need for a patient-centred approach to asthma management in which patients are educated to adjust their medication in a more appropriate manner. This approach, combined with a written personal action plan, has the potential to improve asthma control.

## Competing interests

MRP has received honoraria for lectures, educational activities and ad hoc consultancies from AstraZeneca, Vitaris, GSK and Novartis and research funding from the AstraZeneca Foundation. TvdM has received research grants from AstraZeneca, GSK and Altana, speakers' fees from AstraZeneca, GSK, Altana and MSD, and has participated in advisory boards for Altana, AstraZeneca and Boehringer Ingelheim. SEM received funding for travel to the 2005 European Respiratory Society congress, and honoraria from AstraZeneca for participating in a steering committee and for speaking at a press conference. WWB has received research funds from AstraZeneca, Dynavax, GlaxoSmithKline, Merck and sanofi-aventis. WWB has served on advisory boards for AstraZeneca, Merck, Pfizer and sanofi-aventis, has been a consultant to Dynavax, Schering, Merck and Wyeth, and a member of a steering committee for GlaxoSmithKline.

## Authors' contributions

All authors formed the INSPIRE steering committee and were involved in developing the questionnaire. M. R. Partridge contributed to the conception and design of the study, the analysis and interpretation of the data, and the drafting of the paper. T. van der Molen, S.E. Myrseth and W. W. Busse contributed to the design of the study and to revising the paper critically. All authors made final decisions on all aspects of the article and hence are in agreement with, and approve, the final version of the submitted article.

## Pre-publication history

The pre-publication history for this paper can be accessed here:



## Figures and Tables

**Table 1:** Patient demographics and characteristics

Characteristic	Patients (n = 3415)
Male, %	35
Mean age ± SD, years	45.2 ± 16.7
Recruitment of patients*, n (%)	
PCP	2600 (76)
Specialist	785 (23)
Mean asthma duration, years	16.0
Current smokers, n (%)	714 (21)
Daily maintenance therapy prescribed, n (%)^†^	
ICS no LABA	1018 (30)
ICS plus LABA	2393 (70)
Budesonide/formoterol	769 (23)
Salmeterol/fluticasone	1305 (38)
Self-assessed overall health when feeling well, n (%)	
Very good	1627 (48)
Good	1221 (36)
Average	431 (13)
Bad	87 (3)
Very bad	49 (1)
Self-assessed asthma level over previous 7 days versus general level^‡^, n (%)	
Relatively good	2498 (73)
Relatively bad	871 (26)

*Definition of abbreviations: *ICS=inhaled corticosteroids; LABA=long-acting β_2_-agonist;

SD=standard deviation.

*Data not available for 30 patients. ^†^Data not available for 4 patients. Data not available for 46 patients.

**Table 2:** Patients' (n = 3415) attitudes to asthma management (scale: disagree strongly, disagree somewhat, agree somewhat, agree strongly [data for patients who agreed with each statement are presented])

Attitude statement	Agree somewhat n (%)	Agree strongly n (%)
I want to take treatments that provide immediate relief	577 (17)	2498 (73)
I use my medication as and when necessary	530 (16)	2240 (66)
I am confident I know my asthma well enough to intervene early to try and prevent worsening symptoms	829 (24)	2069 (61)
I prefer to adjust my maintenance ICS/combination medication to the changes of my asthma, taking less when feeling well and more when feeling worse	624 (18)	1723 (50)
I am concerned about taking too much medication when I am well	719 (21)	1139 (33)
Despite taking my medication as my doctor tells me to, I still have a fear of having a serious asthma attack	682 (20)	1000 (29)
When I feel well, I believe there is no need to take my medication every day	464 (14)	837 (25)
I would prefer to take a high dose of ICS/combination medication to try to avoid as many symptoms as possible	588 (17)	626 (18)
I am much more likely to try to manage my asthma myself, rather than visit my physician as soon as my symptoms become bothersome	804 (24)	1601 (47)
I am concerned about taking higher doses of medication due to possible side effects	792 (23)	1246 (36)

**Table 3:** Proportion of patients reporting that worsening asthma limited/prevented their daily activities

Activity	No. of patients (%) (n = 3415)
Exercise and physical activity	2476 (73)
Leisure activities	1671 (49)
Social commitments	1340 (39)
Intimacy with partner	999 (29)
Work	973 (28)
Time spent with family	535 (16)

**Table 4:** Patients' reported worst drawbacks of having asthma (scale: disagree strongly, disagree somewhat, agree somewhat, agree strongly [data for patients who agreed with each statement are presented])

Drawback	No. of patients (%) (n = 3415)
The panic I feel when symptoms start to become bothersome	2477 (73)
The interference with my daily life	2490 (73)
The thought of having to go to the emergency room/hospital	1835 (54)
Having a sudden attack in front of my children, friends and others	1751 (51)
The thought of feeling different from other people	948 (28)
